# Evaluation of Wegener’s granulomatosis using 18F-fluorodeoxyglucose positron emission tomography/computed tomography

**DOI:** 10.1007/s12149-012-0675-3

**Published:** 2012-12-16

**Authors:** Kimiteru Ito, Ryogo Minamimoto, Hiroyuki Yamashita, Setsuko Yoshida, Miyako Morooka, Momoko Okasaki, Akio Mimori, Kazuo Kubota

**Affiliations:** 1 Department of Radiology, National Center of Neurology and Psychiatry, 4-1-1 Ogawahigashi-cho, Kodaira, Tokyo 187-8551 Japan; 2 Division of Nuclear Medicine, National Center for Global Health and Medicine, Tokyo, Japan; 3 Division of Rheumatic Diseases, National Center for Global Health and Medicine, Tokyo, Japan; 4 Department of Internal Medicine, Kawakita General Hospital, Tokyo, Japan

**Keywords:** Wegener`s granuloma, Vasculitis, ANCA, PET/CT, FDG

## Abstract

**Objective:**

Wegener’s granulomatosis (WG) is a relatively rare disease characterized by granulomatous necrotizing vasculitis that primarily involves small- and medium-sized vessels. Systemic findings observed on ^18^F-fluorodeoxyglucose (FDG) positron emission tomography (PET)/computed tomography (CT) have not been well reported. The purpose of this study was to evaluate the FDG PET/CT imaging in the diagnosis and follow-up of patients with WG.

**Materials and methods:**

Thirteen FDG PET/CT images obtained for 8 patients (2 men and 6 women) with WG were retrospectively analyzed. Of these, 6 were performed for diagnosis, 2 for restaging and follow-up, and 5 for assessment of treatment efficacy. Maximum standardized uptake values (max SUVs) and visual analyses were used to interpret the FDG PET/CT images. In addition, nonenhanced CT findings obtained during FDG PET/CT were described.

**Results:**

WG lesions of the upper respiratory tract and lung were more clearly detected by FDG PET/CT fusion imaging than by nonenhanced CT alone, and all of the active lesions showed decreased FDG uptake after treatment. In addition, FDG PET/CT can provide complementary information to indicate biopsy site based on FDG uptakes.

**Conclusions:**

FDG PET/CT is a feasible modality for evaluating lesion activities, therapeutic monitoring, and follow-up of WG. Furthermore, biopsy sites of WG lesions may be determined by FDG PET/CT.

## Introduction

Wegener’s granulomatosis (WG) is a relatively rare disease characterized by granulomatous necrotizing vasculitis that primarily involves small- and medium-sized vessels. WG was initially described as a rhinogenic granulomatous disease by Friedrich Wegener in 1936 [1]. It usually affects middle-aged individuals, (average age, 50 years), and males and females are affected at equal rates. The majority of clinical cases are seen in Caucasians [2]. No specific etiology has been identified to date, although WG etiology is associated with the presence of circulating antineutrophil cytoplasmic antibodies (ANCA) directed against proteinase 3 (PR3) or myeloperoxidase (MPO) [3, 4]. Untreated patients rapidly develop severe inflammation and renal failure, which can prove life threatening if not treated on time. Early diagnosis is, therefore, important for the improvement of prognosis.

Computed tomography (CT) findings for the diagnosis of WG have been reported [5–7]. Recent case reports have documented 18F-fluorodeoxyglucose (FDG) positron emission tomography (PET) findings in patients with WG. These suggest that WG exhibits relatively high FDG uptake before treatment, which promptly decreases after treatment [8–12]. However, no organized studies on the use of FDG PET/CT for patients with WG have been reported. The purpose of this retrospective study was to evaluate the role of FDG PET/CT in the diagnosis and follow-up of a series of WG patients.

## Materials and methods

### Patients and laboratory data

FDG PET/CT scans performed between April 2005 and March 2011 at our hospital were retrospectively analyzed, and 13 FDG PET/CT images of 8 patients (2 men and 6 women; age 39–86 years; mean age 69.8 years) with WG were selected for analyses. On the basis of clinical and/or biopsy findings, all patients were diagnosed with WG on the basis of the 1990 American College of Rheumatology criteria [13]. The results of several laboratory parameters measured within 2 weeks of initial FDG PET/CT, including white blood cell count (WBC), hemoglobin (Hgb), C-reactive protein level (CRP), serum creatine, serum blood urea nitrogen (BUN), PR3-ANCA titer, and MPO-ANCA titer, were examined. Informed consent was waived because of retrospective analysis. This study was approved by the Institutional Review Board of our hospital.

### FDG PET/CT technique and evaluation

Each patient was instructed to fast for more than 6 h, following which his/her blood glucose level was measured. Subsequently, 185–370 MBq of FDG was administered. An hour after injection, FDG images were acquired from the skull vertex to the upper thighs. All studies were performed using combined PET/CT tomography (Biograph sensation 16; Siemens). Emission scans were acquired for 3 min per bed position. An ordered-subset expectation maximization algorithm (3 iterations, 8 subsets) was used to reconstruct all PET data. Findings of CT performed without contrast medium (nonenhanced CT) during FDG PET/CT (60 mAs; 120 keV; section width, 5 mm; 0.5 s/CT rotation) were used for attenuation correction and identification of anatomical localization.

Two board-certified radiologists/nuclear medicine physicians used visual assessments separately. One of the physicians measured the standardized uptake values (SUVs). Regions of interest (ROIs) were drawn to encompass the original location of WG lesions. Maximum SUVs (max SUVs) in the ROIs were then recorded. For semiquantitation, a dedicated work station was used to obtain max SUVs in each ROI.

Findings of nonenhanced CT performed during FDG PET/CT were used for evaluation of the morphology of the upper respiratory tract and pulmonary lesions (e.g. shape, location, and size). The two evaluators assessed these CT findings using visual assessment.

### Statistical analysis

Continuous variables that may not have been normally distributed because of the small number of patients or examinations were reported as median and interquartile ranges. Spearman’s correlation coefficients (two-tailed) were used to evaluate whether laboratory data correlated with max SUVs. A *p* value of <0.05 was considered statistically significant. SPSS ver. 18.0 for Windows (SPSS Japan Inc, Japan) was used for all statistical analyses.

## Results

The patients’ background characteristics and laboratory data are shown in Table 1. Two of the 8 patients had a past history of prednisone and immunosuppressant therapy. In all but 2 patients, the WG lesions were located in the upper respiratory tract, lungs, or kidneys. The MPO- and PR3-ANCA levels were abnormal in 4 patients. No significant correlation was found between max SUVs and the laboratory data (WBC, Hgb, CRP, creatine, BUN, PR3-ANCA titer, and MPO-ANCA titer). In all patients except 1, histological findings were indicative of vasculitis. One patient (Case7) was diagnosed with WG based on the symptoms of upper respiratory tract and chest X-ray findings because she rejected to undergo a biopsy. At the end of the observation period, all the patients were in remission, and 2 transferred to other hospitals.

**Table 1:** Patient characteristics and laboratory data

Patient No	Sex	Age	Purpose of the study	General symptom	Clinical findings in upper respiratory tract	Clinical findings in lung	Clinical findings in kidney	Biopsy site	PR3- ANCA	MPO- ANCA	WBC	Hgb	CRP	Cr	BUN
1	F	67	Initial	Fever, LN swelling	No symptom	No symptom	RPGN	Nasal biopsy	2.4	204.0	10380	9.6	17.39	0.56	7.8
2	F	67	Initial	Fever, arthritis	Exudative otitis media, sinusitis,	Cough	Hematuria, RPGN	VATS	<1.3	9.6	8340	10.8	7.13	0.6	7.2
3	F	65	Initial	Fever, headache	Exudative otitis media, episcleritis	Cough	RPGN	VATS, bone biopsy	5.5	32.4	6300	10.4	11.67	0.57	8.3
4	M	84	Maintanence	Fever	No symptom	Cough	Chronic failure	Nasal and kidney biopsy	<1.3	N.A.	3980	11.0	1.46	0.51	14.5
5	F	39	Maintanence	Fever	No symptom	No symptom	RPGN	Vascular angitis nasal biopsy	1.5	<1.3	7710	11.4	0.91	0.99	22.8
6	F	79	Initial	Fever	No symptom	No symptom	RPGN	Vascular angiitis nasal biopsy	<1.3	36.0	12200	10.0	0.31	2.68	61.6
7	F	82	Initial	Fever	Exudative otitis media, facial nerve palsy	Cough	No symptom	Not-conducted	<1.3	70.2	28270	10.3	16.15	0.28	25.2
8	M	76	Initial	Fever	Nasal bleeding	No symptom	No symptom	Vascular angiitis nasal biopsy	<1.3	<1.3	6090	11.9	0.49	0.94	17.1

*RPGN* rapidly progressive glomerulonephritis, *ANCA* antineutrophil cytoplasmic antibodies, *MPO* myeloperoxidase (<2.0 U/ml), *PR-3* proteinase 3 (<3.5 U/ml), *VATS* video-assisted thoracic surgery, *WBC* white blood cell count (3500–8500/μl), *Hgb* hemoglobin (11.5–15.0 g/dl), *CRP* C-reactive protein (<0.3 mg/dl), *Cr* creatinine (0.4–0.7 mg/dl), *BUN* blood urea nitrogen (8.0–22.0 mg/dl), *NA* not available

A total of 13 FDG PET/CT scans of 8 patients were analyzed. The subsets of patients were examined for different purposes, which are listed in Table 2 and described below. The reviewers completely agreed in visual interpretation and max SUVs. They then evaluated the shape and location of WG lesions on nonenhanced CT during FDG PET/CT using visual analysis (Table 3). In 5 patients who underwent PET/CT examinations before and after treatment, all lungs and upper respiratory lesions had shrunk in size.

**Table 2:** Indications and imaging characteristics of ^18^F-fluorodeoxyglucose (FDG) positron emission tomography/computed tomography

No	Indications for PET/CT	BG	max SUVs for nasal, throat, and ear lesions	max SUVs for lung lesions	Abnormal FDG uptake in other areas
1	Diagnosis	88	8.43	7.10	Bone marrow, mediastinal and hilar LNs, and spleen
	Response	139	1.89	2.07	No finding
2	Diagnosis	76	2.54	6.79	Mediastinal and hilar LNs
3	Diagnosis	85	4.30	3.41	Sacrum and spine
	Response	85	1.41	1.11	Esophagus and left hip joint
4	Follow-up	96	1.63	1.27	Esophageal cancer
5	Relapse	82	4.18	1.41	Stomach
6	Diagnosis	98	5.10	4.00	Decreased renal uptake due to renal failure
	Response	184	1.62	2.12	Muscle uptake due to type 2 DM, decreased renal uptake due to renal failure
7	Diagnosis	123	5.47	0.76	Bone marrow, spleen
	Response	85	1.46	0.97	Esophagus, left pubis (fracture)
8	Diagnosis	128	7.89	0.69	Right lobe of prostate
	Response	132	1.37	0.58	Right lobe of prostate

*BG* blood glucose, *LN* lymph node, *DM* diabetes mellitus

**Table 3:** Nonenhanced CT findings during ^18^F-fluorodeoxyglucose (FDG) positron emission tomography (PET)/computed tomography (CT)

No	Lesion characteristics and location on nonenhanced CT performed during FDG PET/CT					
	Nasal, throat, and ear lesions		Lung lesions		Kidney lesions	Others
	Uptake location	Shape on CT	Uptake location	Shape on CT	Shape on CT	Shape on CT
1	Nasal septum	Mild mucosal thickness	Bilateral lower lobes	Bronchial wall thickness, and consolidations	No findings	No findings
2	Nasal septum	Not detected	Both lungs	Multiple nodules and consolidations	No findings	No findings
3	Bilateral auditory tube	Not detected	Bilateral upper lobes and right lower lobe	Multiple nodules, consolidation, and pleural effusion	No findings	Soft tissue density in sacrum
4	No findings	No findings	No findings	Fibrosis and small nodules in the right middle and lower lobes	No findings	Esophageal cancer
5	Right maxillary sinus	Destruction of right maxillary wall, soft tissue of right orbit	No findings	Atelectasis of left upper lobe bronchial wall thickness,	No findings	No findings
6	Nasal septum	Mild mucosal thickness	Bilateral lower lobes	Consolidation, IP	No findings	GB stones
7	Bilateral auditory tube	Not detected	Bilateral lungs	Bronchitis	No findings	Compression fractures
8	Right maxillary sinus	Mild mucosal thickness	No lesion	No lesion	No findings	GB stones

*GB* gallbladder, *IP* interstitial pneumonia

### Diagnosis and initial activity

**Fig. 1:**
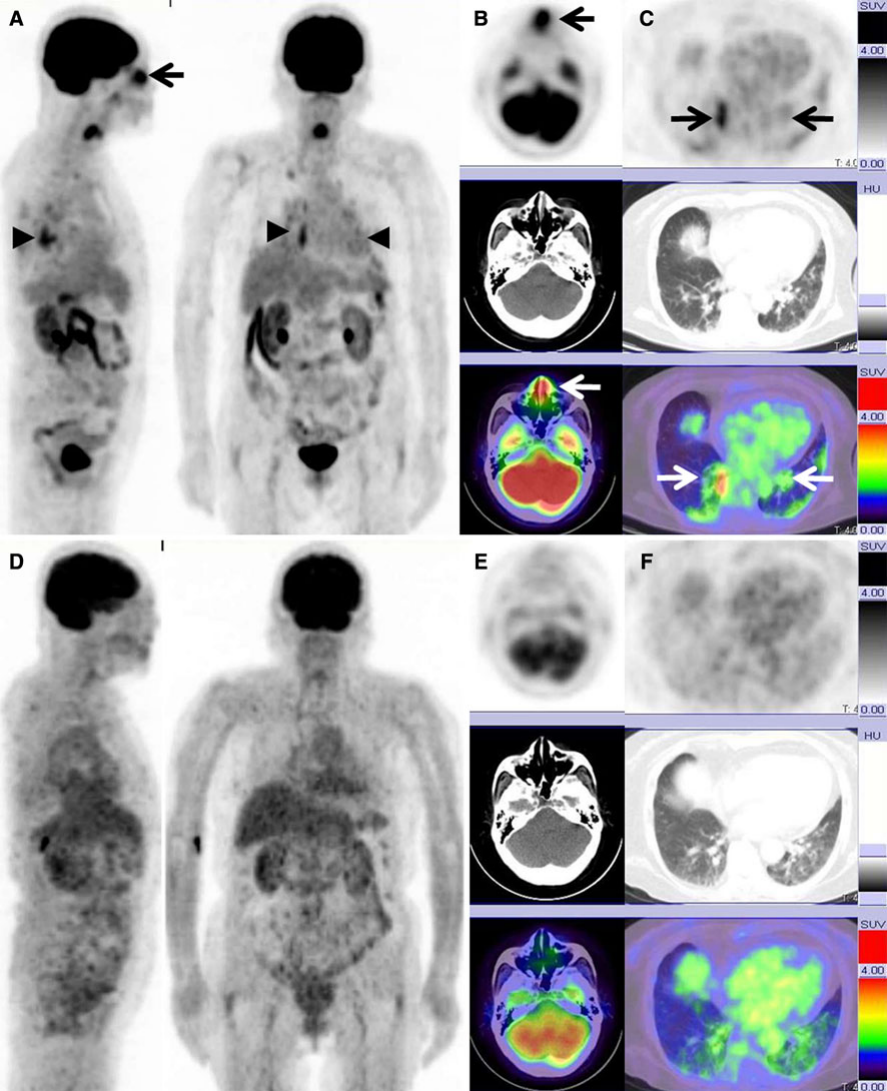
A 79-year-old woman (Case 6) underwent FDG PET/CT after developing a fever of unknown origin. Serum MPO-ANCA titer was elevated to 295 U/ml, although serum PR3-ANCA titer was within normal limits. After a biopsy performed from nasal FDG uptake area, she was diagnosed with WG. **a** A maximum intensity projection (MIP) image showing abnormal FDG uptake in the nasal mucosa (*arrow*), bilateral lungs (*arrow heads*), and decreased renal uptake due to renal failure. **b** An axial FDG PET/CT image showing increased FDG uptake in the nasal wall (arrow; max SUV, 5.10), although the findings of nonenhanced CT alone do not show the nasal lesion. **c** An axial FDG PET/CT image showing increased FDG uptake at the nodular shadows in both lungs (*white and black arrows*; max SUV, 4.00). After the predonisolone and immunosuppressant therapy, FDG PET/CT was performed to survey the malignancy. The patient had type 2 diabetes mellitus due to steroid therapy. **d** MIP images obtained after treatment show no FDG uptake in the nasal mucosa and mild FDG uptake in bilateral lungs. **e**, **f** Axial FDG PET/CT images obtained after treatment show no FDG uptake in the nasal mucosa (max SUV, 1.62) and lungs (max SUV: 2.12). The both lung lesions resisted antibiotic therapy. Finally we diagnosed these lung opacities as one of the findings on WG on the basis of the treatment response

**Fig. 2:**
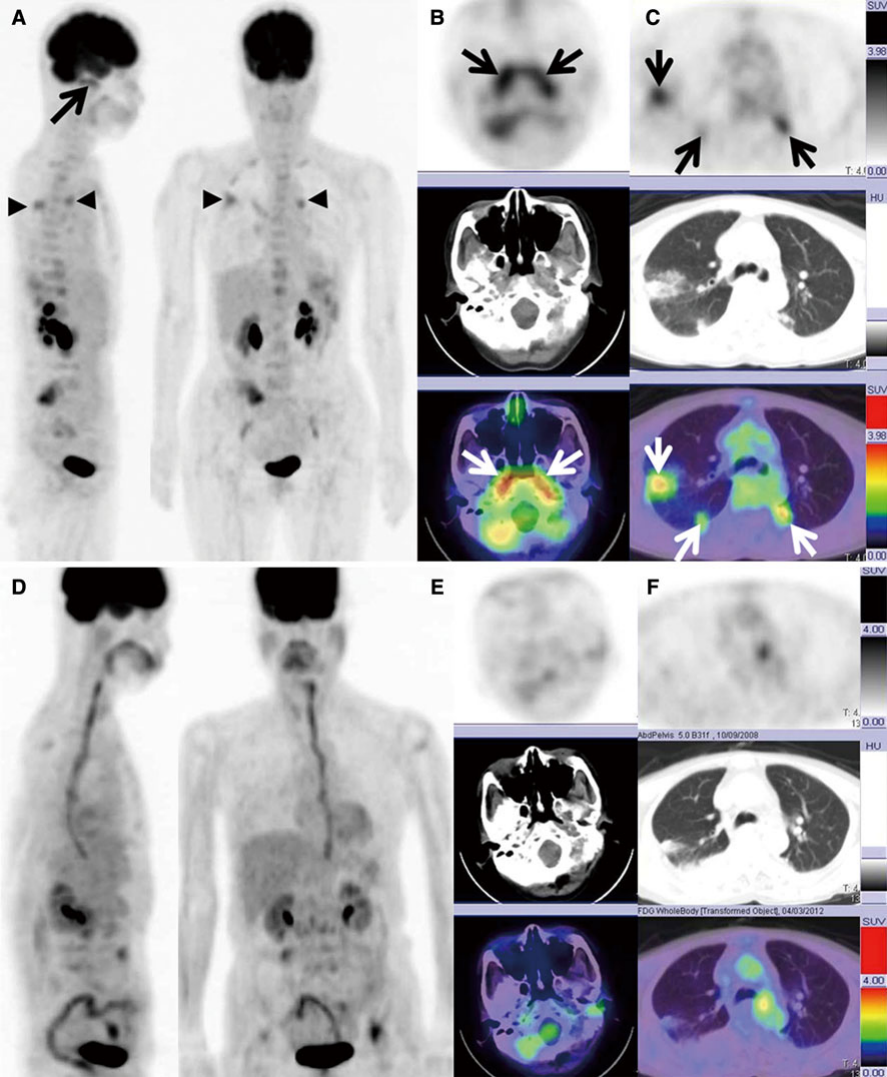
A 65-year-old woman (Case 3) with WG underwent PET/CT. Serum MPO-ANCA titer and PR3-ANCA titer were elevated to 27.4 and 5.5 U/ml, respectively. **a** An MIP image showing the abnormal FDG uptake in the nasopharynx (*arrow*) and lung (*arrow heads*). **b** An axial FDG PET/CT image showing increased FDG uptake in the bilateral auditory tubes (*white and black* *arrows*; max SUV, 4.30), although the findings from nonenhanced CT alone do not show abnormality in the bilateral auditory tubes. **c** An axial FDG PET/CT image showing increased FDG uptake at the nodular shadows in both lungs (*white and black arrows*; max SUV, 3.41; *white arrows*). At follow-up FDG PET/CT performed 5 months after treatment, both serum MPO-ANCA and PR3-ANCA titers were within normal limits. **d** Five months after treatment, an MIP image shows no FDG uptake in the nasal mucosa and bilateral lungs (*white arrows*). **e**, **f**. Five months after treatment, an axial FDG PET/CT image shows no FDG uptake in the nasal mucosa (max SUV, 1.41) and bilateral lungs (max SUV, 1.11) (*white arrows*)

Of the total, 6 patients (Nos. 1, 2, 3, 6, 7, and 8) were examined by FDG-PET/CT to diagnose. FDG uptake revealed thickening of the nasal mucosa in 2 patients (Fig. 1) and exudative otitis media in 3 patients (Fig. 2), 4 of whom had lesions that were hardly detected by CT imaging alone. The interquartile range of max SUV for the 6 patients with upper respiratory tract lesions and 4 with lung lesions of the 6 patients was 3.86–8.03 (median 5.28) and 3.56–7.02 (median 5.39), respectively. Based on FDG uptakes, Nos. 1, 3, 6 and Nos. 2, 3 were biopsied at the upper respiratory tract and the lung, respectively. However, kidney lesions could not be detected by FDG PET/CT. All patients were treated with prednisone and immunosuppressant therapy after diagnosis.

### Treatment response evaluation

Of the total, 5 patients (Nos. 1, 3, 6, 7, and 8) underwent FDG PET/CT for evaluating the efficacy of treatment. The median and interquartile range of the interval between pre- and post-treatment FDG PET/CT were 12 months and 3–34 weeks, respectively. Shortest interval between pre- and post-treatment FDG PET/CT was 1 month (Case 1). All patients had received chemotherapy for WG. Five FDG-avid lesions in upper respiratory tract completely disappeared after treatment. Five FDG-avid lesions in both lungs were found in 5 patients before treatment. Of these lesions, 3 showed background level FDG uptake, and 2 showed lower FDG uptake compared with blood pool or mediastinal uptake after treatment. The interquartile range of max SUV for the 5 patients with upper respiratory tract lesions and 3 with lung lesions of the 5 patients was 1.39–1.75 (median 1.46) and 1.11–2.07 (median 2.07), respectively.

### Follow-up

Two patients who had been diagnosed with WG (Nos. 4 and 5) were examined by FDG PET/CET to evaluate exacerbations of WG lesions based on physical or other imaging modality findings during follow-up after treatment completion. FDG PET/CT was performed in 1 patient (No. 5) to evaluate WG activity after treatment because she developed fever. The other patient (No. 4) underwent FDG PET/CT for staging before initiation of chemoradiotherapy for esophageal cancer. These FDG PET/CT images showed no active FDG uptake associated with WG lesions.

## Discussion

WG is a rare type of systemic vasculitis that involves the upper and lower respiratory tracts and the kidneys [1]. There are several reported cases in which active WG lesions were detected by FDG PET [8–12]. The results of our study indicated that WG lesions in the upper respiratory tract were easier to detect by FDG uptake during FDG PET/CT than by nonenhanced CT alone (Figs. 1, 2).

The diagnosis of WG is occasionally delayed because of nonspecific clinical symptoms. The use of CT for the diagnosis of WG has been reported, although specific CT diagnostic findings have not been confirmed because of various lesion patterns [5–7]. A few reports have shown the usefulness of gallium-67 citrate (Ga-67) scintigraphy for the diagnosis and assessment of disease activity in patients with WG [14]. Of late, there have been a few case reports on FDG PET findings in patients with WG [8–12]. FDG PET/CT imaging findings, including max SUV, is useful for diagnosis as well as assessment of treatment efficacy in patients with malignancy and inflammation, and it is reportedly superior to Ga-67 scintigraphy in the field of oncology [15, 16]. Our study also showed FDG PET/CT to be a complementary modality via the indication of suitable biopsy sites and assessment of treatment efficacy. As an additional advantage, nonenhanced CT during FDG PET/CT facilitates the diagnosis of WG by behaving as a complementary modality for detecting lesions on the basis of anatomical location. CT during FDG PET/CT frequently showed residual lesions in the lungs after treatment, and FDG uptake showed that these residual lesions were inactive.

The subjects of this study had relatively low serum PR3-ANCA titers. The correlation between PR3-ANCA titer and WG has been well established [3, 4]. Although PR3-ANCA testing can aid in diagnosis, the findings are not conclusive. Conversely, a negative PR3-ANCA test result is not enough to exclude WG diagnosis, and biopsy remains the standard means of diagnosis [17]. This fact suggests that FDG uptake may become one of the ideal imaging to evaluate WG activity.

The first limitation of this study was the small sample size. However, WG is a rare disease, and opportunities to study FDG PET/CT images of patients with WG are rare. Moreover, this limitation is unlikely to have affected our conclusion because the usefulness of PET for other types of vasculitis has been demonstrated in previous studies. A second limitation is the various intervals between treatment and FDG PET/CT in each patient. However, we were able to perform FDG PET/CT within 1 month after treatment to accurately interpret remission status.

In conclusion, this study showed FDG PET/CT to be a useful modality for diagnosis, assessment of treatment efficacy, and follow-up of WG, even in cases with residual masses. In addition, FDG PET/CT can provide complementary information for the diagnosis and assessment of WG in addition to the information obtained by ANCA titers.

## References

[1] Woywodt A, Matteson EL. Wegener’s granulomatosis–probing the untold past of the man behind the eponym. Rheumatology (Oxford). 2006;45:1303–6. 10.1093/rheumatology/kel258 16887845

[2] Olivencia-Simmons I (2007). Wegener’s granulomatosis: symptoms, diagnosis, and treatment. J Am Acad Nurse Pract..

[3] Uehara A, Hirabayashi Y, Takada H (2008). Antibodies to proteinase 3 prime human oral, lung, and kidney epithelial cells to secrete proinflammatory cytokines upon stimulation with agonists to various Toll-like receptors, NOD1, and NOD2. Clin Vaccine Immunol.

[4] Schönermarck U, Lamprecht P, Csernok E, Gross WL (2001). Prevalence and spectrum of rheumatic diseases associated with proteinase 3-antineutrophil cytoplasmic antibodies (ANCA) and myeloperoxidase-ANCA. Rheumatology (Oxford).

[5] Grindler D, Cannady S, Batra PS (2009). Computed tomography findings in sinonasal Wegener’s granulomatosis. Am J Rhinol Allergy..

[6] Ananthakrishnan L, Sharma N, Kanne JP (2009). Wegener’s granulomatosis in the chest: high-resolution CT findings. AJR Am J Roentgenol.

[7] Martinez F, Chung JH, Digumarthy SR, Kanne JP, Abbott GF, Shepard JA (2012). Common and uncommon manifestations of Wegener granulomatosis at chest CT: radiologic-pathologic correlation. Radiographics..

[8] Watts RA, Al-Taiar A, Scott DG, Macgregor AJ (2009). Prevalence and incidence of Wegener`s granulomatosis in the UK general practice research database. Arthritis Rheum.

[9] Almuhaideb A, Syed R, Iordanidou L, Saad Z, Bomanji J (2011). Fluorine-18-fluorodeoxyglucose PET/CT rare finding of a unique multiorgan involvement of Wegener’s granulomatosis. Br J Radiol.

[10] Nishiyama Y, Yamamoto Y, Dobashi H, Kameda T (2010). Clinical value of 18F-fluorodeoxyglucose positron emission tomography in patients with connective tissue disease. Jpn J Radiol..

[11] Ueda N, Inoue Y, Himeji D, Shimao Y, Oryoji K, Mitoma H (2010). Wegener’s granulomatosis detected initially by integrated 18F-fluorodeoxyglucose positron emission tomography/computed tomography. Mod Rheumatol.

[12] Beggs AD, Hain SF (2002). F-18 FDG-positron emission tomographic scanning and Wegener’s granulomatosis. Clin Nucl Med.

[13] Leavitt RY, Fauci AS, Bloch DA, Michel BA, Hunder GG, Arend WP (1990). The American College of Rheumatology 1990 criteria for the classification of Wegener’s granulomatosis. Arthritis Rheum.

[14] Slart RH, Jager PL, Poot L, Piers DA, Tervaert JW, Stegeman CA (2003). Clinical value of gallium-67 scintigraphy in assessment of disease activity in Wegener’s granulomatosis. Ann Rheum Dis.

[15] Kostakoglu L, Goldsmith SJ (2000). Fluorine-18 fluorodeoxyglucose positron emission tomography in the staging and follow-up of lymphoma: is it time to shift gears?. Eur J Nucl Med.

[16] Kostakoglu L, Leonard JP, Kuji I, Coleman M, Vallabhajosula S, Goldsmith SJ (2002). Comparison of fluorine-18 fluorodeoxyglucose positron emission tomography and Ga-67 scintigraphy in evaluation of lymphoma. Cancer.

[17] Seo P, Stone JH (2004). The antineutrophil cytoplasmic antibody-associated vasculitides. Am J Med.

